# A hybrid multi-particle approach to range assessment-based treatment verification in particle therapy

**DOI:** 10.1038/s41598-023-33777-w

**Published:** 2023-04-25

**Authors:** Ilker Meric, Enver Alagoz, Liv B. Hysing, Toni Kögler, Danny Lathouwers, William R. B. Lionheart, John Mattingly, Jasmina Obhodas, Guntram Pausch, Helge E. S. Pettersen, Hunter N. Ratliff, Marta Rovituso, Sonja M. Schellhammer, Lena M. Setterdahl, Kyrre Skjerdal, Edmond Sterpin, Davorin Sudac, Joseph A. Turko, Kristian S. Ytre-Hauge, The NOVO collaboration

**Affiliations:** 1 Department of Computer Science, Electrical Engineering and Mathematical Sciences, Western Norway University of Applied Sciences, P.O. Box 7030, 5020 Bergen, Norway; 2 Department of Oncology and Medical Physics, Haukeland University Hospital, Bergen, Norway; 3 Department of Physics and Technology, University of Bergen, P.O. Box 7803, 5020 Bergen, Norway; 4 OncoRay–National Center for Radiation Research in Oncology, Faculty of Medicine and University Hospital Carl Gustav Carus, Technische Universität Dresden, Helmholtz-Zentrum Dresden-Rossendorf, Dresden, Germany; 5 Helmholtz-Zentrum Dresden-Rossendorf, Institute of Radiooncology–OncoRay, Dresden, Germany; 6 Delft Universiy of Technology, Delft, The Netherlands; 7 The University of Manchester, Manchester, UK; 8 Department of Nuclear Engineering, North Carolina State University, Raleigh, NC USA; 9 Ruder Boskovic Institute, Zagreb, Croatia; 10 Target Systemelektronik GmbH & Co. KG, Wuppertal, Germany; 11 Holland Particle Therapy Centre, Delft, The Netherlands; 12 Department of Oncology, Laboratory of Experimental Radiotherapy, KU Leuven, Leuven, Belgium

**Keywords:** Imaging techniques, Biomedical engineering, Three-dimensional imaging, Whole body imaging

## Abstract

Particle therapy (PT) used for cancer treatment can spare healthy tissue and reduce treatment toxicity. However, full exploitation of the dosimetric advantages of PT is not yet possible due to range uncertainties, warranting development of range-monitoring techniques. This study proposes a novel range-monitoring technique introducing the yet unexplored concept of simultaneous detection and imaging of fast neutrons and prompt-gamma rays produced in beam-tissue interactions. A quasi-monolithic organic detector array is proposed, and its feasibility for detecting range shifts in the context of proton therapy is explored through Monte Carlo simulations of realistic patient models and detector resolution effects. The results indicate that range shifts of ${1}\,\hbox {mm}$ can be detected at relatively low proton intensities ($22.30(13)\times 10^{7}$ protons/spot) when spatial information obtained through imaging of both particle species are used *simultaneously*. This study lays the foundation for *multi-particle* detection and imaging systems in the context of range verification in PT.

## Introduction

Particle therapy (PT) is an emerging radiotherapy (RT) modality for treatment of cancer, with increasing numbers of patients receiving PT as part of their treatment course. As of the end of 2021, about 325,000 cancer patients have been treated in about 116 clinical PT facilities worldwide. The current trend is likely to continue in the foreseeable future as more facilities are either under construction (36 PT centers) or are in the planning stage (32 centers) worldwide^1^.

The advantage of PT compared to the state-of-the-art RT with photons is attributable to the physical properties of charged particles where the ionization density is highest within a narrow range of depth, i.e. the Bragg peak. The sharp dose fall-off at the distal end of the Bragg peak is one of the major motivations for treating cancer with PT, as it allows for more conformal dose distributions that spare healthy tissue and thereby reduce adverse effects. However, in practice, uncertainties in the exact positioning of the Bragg peak preclude the use of the sharp distal dose fall-off. These uncertainties, referred to as *range uncertainties*, arise from many different sources such as anatomical and density changes in the non-static patient and uncertainties associated with the estimation of stopping power in tissue^2^. The main motivation for developing systems for real-time range monitoring in PT is therefore to mitigate such uncertainties and thereby unleash the full potential of PT.

Robust treatment planning is the current approach to reduce adverse effects of range uncertainties^3^. This ensures that the prescribed dose is delivered to the tumour, but at the expense of an extra dose to the surrounding healthy tissue. In addition, selection of beam angles is limited to avoid the risk of overshoots to high-risk organs^4,5^. Thus, it is highly desirable to increase the accuracy and adaptation potential of PT, especially for moving tumors and patients with implants or target regions affected by anatomical changes. Ideally, this would be achieved through treatment verification where the dose delivered by the incident particle beam—or at least the range of the therapeutic particle beam—is monitored during treatment. For pencil beam scanning, measurements would ideally be performed for each particle beam spot individually.

During PT, particles are fully stopped inside the patient and cannot be used for monitoring purposes. However, several production channels contribute to the generation of secondary particles: (1) electrons that contribute to the local dose deposition; (2) collisions with target nuclei leaving them excited, followed almost instantly by the emission of high-energy gamma rays (prompt-gamma rays, PG); (3) collisions with target nuclei that directly eject nuclear constituents such as protons (mostly stopped inside the patient) and fast neutrons (FN, mostly escaping the patient).

Devices detecting PGs to estimate their production location have been evaluated in clinical settings^6,7^; they are commonly based on a one-dimensional slit collimation^8,9^. Systems based on the Compton camera principle have also been proposed^10,11^, where successive interactions of the PG in absorber and scatter layers of a detector lead to event cones, enabling two- or three-dimensional reconstruction of the production sites. Furthermore, PG timing and spectroscopy systems have been proposed and tested under clinical conditions^12,13^.

While the use of PGs as potential range probes has been explored extensively, the same is not true for secondary FNs. In a recent study by Marafini et al.^14^, detection and imaging of FNs have been proposed to assess the neutron dose distribution in the patient. In another study by Ytre-Hauge et al.^15^, the use of a proton recoil telescope setup has been proposed to facilitate secondary FN detection and to use the available information for range verification in proton therapy. In another recent study, Lerendegui-Marco et al.^16^ discuss the combined measurements of PGs and thermal/epi-thermal neutrons with a pinhole camera. The study provides, however, no quantifiable insights into the potential benefits for range assessment purposes.

An important point is that an overwhelming majority of the state-of-the-art systems intended for in-vivo particle beam range monitoring rely completely on the detection of a single particle species with resulting limitations in counting statistics, making these systems essentially incapable of monitoring the range of mono-energetic pencil beam spots comprising a limited number of protons (about $10^6$ protons/spot to $10^8$ protons/spot) only^17^. Only very recently, the PT community started shifting its focus to multi-modal detection systems^18,19^ without regards to more compact, hybrid systems that can be used for detection and imaging of multiple particle species simultaneously, reducing overall complexity and the clinical footprint.

**Figure 1:**
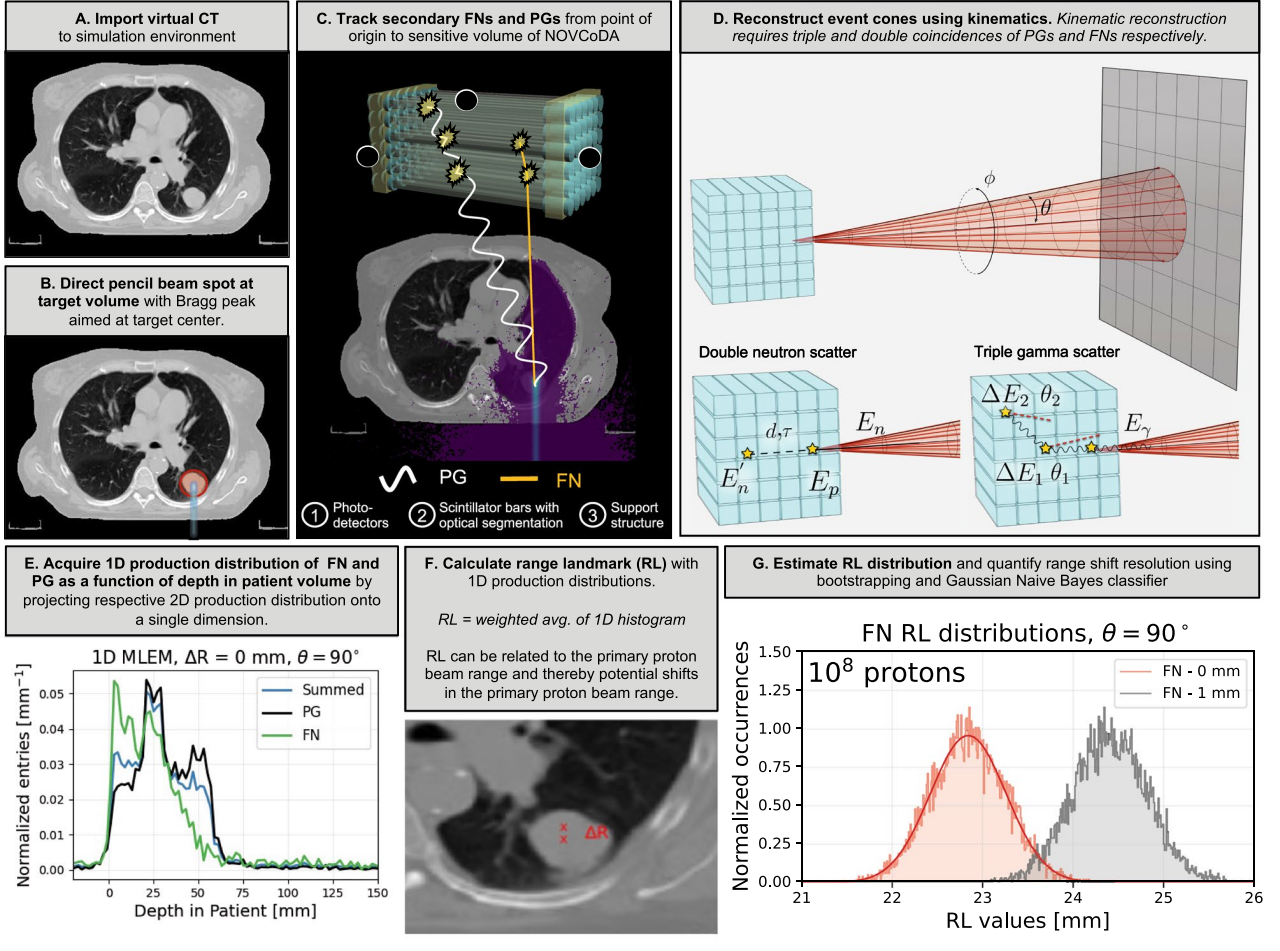
An illustration of the conceptual NOVCoDA design and the analysis workflow. (**A**) A virtual 3D patient model (a patient with non-small cell lung cancer) is imported into the MC simulation environment. (**B**) A mono-energetic proton pencil beam is then directed at the target volume. (**C**) Secondary FNs and PGs are tracked from their points of origin, through the patient geometry and the sensitive volume of the NOVCoDA. Also shown is the conceptual design of the NOVCoDA. (**D**) To enable kinematic reconstruction of event cones, triple and double coincidences are required for PGs and FNs, respectively. Event cones are back projected onto a plane coinciding with the proton beam axis. The back projection is followed by image reconstruction resulting in 2D FN and PG production distributions. (**E**) The 2D production distributions are projected onto a single dimension resulting in 1D histograms of FN and PG distributions as a function of depth in the patient geometry, i.e. along the primary proton beam direction. (**F**) The 1D distributions are then used to calculate a range landmark (RL) parameter that can be related to the primary proton beam range and thereby potential range shifts. (**G**) A bootstrapping procedure combined with a Gaussian Naive Bayes classifier is implemented to estimate the distributions of the calculated RLs and quantify range-shift-resolving capabilities of the NOVCoDA.

In view of the above observations, and to the best of our knowledge, the current literature contains no discussion of hybrid systems combining measurements of PGs and FNs for the purposes of range probing in PT. To this end, the NOVO (NeutrOn and gamma-ray imaging for real-time range VerificatiOn and image guidance in particle therapy) collaboration proposes a novel technology that provides hybrid imaging of PG and FN emissions in PT. The proposed system is expected to provide a sufficiently high sensitivity to FNs and PGs as well as a much smaller footprint than the other state-of-the-art systems. The main building block of the envisaged NOVO system is a compact detector array (NOVCoDA) consisting of densely stacked, optically segmented organic scintillator bars with dual-ended light readout (see Fig. 1 for a conceptual design). For improved sensitivity and enhanced imaging capabilities, the NOVCoDA will make use of novel organic scintillators, such as organic glass scintillators (OGS), with excellent pulse shape discrimination capabilities and time, energy, and position resolutions^20,21^. Furthermore, light readout will be based on state-of-the-art silicon photomultiplier arrays, resulting in a compact design with fast timing and a high number of single avalanche photodiodes. In the prototyping stage, the data acquisition will be performed using commercial fast (500 million samples/s) and high resolution (14-bit) digitizers supplied with custom front-end electronics for signal shaping and amplification. Imaging of incident FNs will rely on double coincident elastic scatters on hydrogen nuclei, i.e. (n,p) scatters. The time-of-flight information between the detector segments will be used for determining the energies of scattered neutrons. For PGs, the imaging will rely on triple coincident scatter events that occur in distinct scintillator bars.

In the current study, we report on the feasibility of a typical NOVCoDA with total volume of $30 \times 20 \times 20$ $\hbox {cm}^{3}$ (a total mass of only about 12kg for the scintillators) in a proton therapy scenario at three different orientations (anterior, $\theta = 0^{\circ }$; oblique, $\theta = 45^{\circ }$; and lateral, $\theta = 90^{\circ }$) of the NOVCoDA with respect to the incident proton beam direction, where the angle, $\theta$, is defined between beam axis and the line connecting the treatment isocenter with the center of the NOVCoDA array. At this initial stage, we base our observations on Monte Carlo (MC) radiation transport models. As a novelty in the methodology, however, we implement a virtual 3D CT scan of a real patient to determine expected sensitivities of the range monitoring. For this purpose, pencil beam spots comprising a defined number of protons of given energy are modeled to enter the patient model, while the production of secondary particles and their possible interactions with the NOVCoDA are simulated. Controlled range shifts, referred to as *true range shifts* in the remainder of this work, are introduced by reducing or increasing the tissue density in masked areas of the patient model. More importantly, the use of a CT-scan of a patient model ensures that tissue heterogeneity is an integral part of the subsequent analysis, including image reconstruction, which is a lacking aspect in most similar studies. The methods applied in the current study are illustrated in Fig. 1. It should be stressed that in this work, only 3D patient models were used. The corresponding simulations were also executed in 3D. For the ground truth data, the 3D production coordinates of FNs and PGs were projected onto a single dimension resulting in 1D histograms, referred to as 1D distributions. Exact production coordinates of FNs and PGs cannot, however, be reconstructed. Only voxel-wise (in 3D) or pixel-wise (in 2D) probability distributions can be reconstructed. In this work, the reconstruction was performed in 2D by back projection of FN and PG event cones onto a plane that coincides with the proton beam axis. The reconstructed 1D FN and PG distributions were obtained by taking a slice through the reconstructed 2D image.

The purpose of the current study is therefore twofold: (1) investigations of the feasibility of using the NOVCoDA to detect controlled range variations in a virtual 3D patient model, and to determine expected sensitivities as a function of the required number of protons for mono-energetic proton beam spots and (2) investigations of the potential applicability of the NOVCoDA to detect treatment deviations in an example, realistic patient case with a locally advanced non-small cell lung cancer, using FN data only, PG data only, as well as the sum of FN and PG data. For both, true collision parameters obtained directly from MC models and collision parameters accounting for detector resolution effects were used. When using true collision parameters, the sensitivities and the capabilities concerning identification of realistic range shifts are reported for all three orientations of the NOVCoDA whereas only the lateral orientation is considered for the data sets incorporating detector resolution. This is justified by the fact that FNs are mostly emitted in the forward direction^15,22^, i.e. along the incident proton beam direction, and that flexible positioning of an on-line imaging device might be necessary to enable monitoring of different treatment sites with various beam configurations^23^.

## Results

### FN and PG production yields and their dependence on introduced range shifts

**Figure 2:**
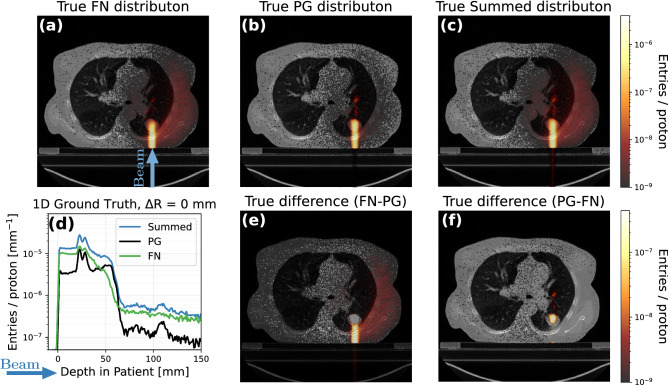
2D and 1D ground truth distributions of detected FNs and PGs. (**a**, **b**) 2D ground truth distributions of successfully detected FNs and PGs obtained for the laterally positioned NOVCoDA ($\theta = 90^{\circ }$). Also shown are the combined distributions (**c**), the difference between FN and PG distributions (**e**), the difference between PG and FN distributions (**f**) as well as a 1D projection of the production distributions of detected FNs and PGs along the incident proton beam axis (**d**). Clearly, both the 1D and 2D the distributions indicate that FNs predominate the overall response at shallower depths whereas PGs predominate near the end of range of the incident proton beam.

Firstly, for any imaging device used for the purposes of range assessment, the expected relation between signals extracted from the device and true range shifts must be quantified. In the MC simulations, the NOVCoDA was placed anterior, obliquely and laterally to the patient model, and the overall response to incident FNs and PGs was examined in all three orientations. Successful detection of an FN event required the incident neutron to collide elastically on hydrogen nuclei at least twice, whereas a successfully detected PG event required the incident gamma ray to scatter at least three times in the sensitive volume of the NOVCoDA, where the third scatter can be absorption or another scatter. To obtain *ground truth* distributions, the true 3D spatial coordinates of where successfully detected FNs and PGs originated from within the patient model were then obtained directly from phase-space files generated in MC simulations. Representative two-dimensional as well as one-dimensional projections of FN, PG and the summed production yields are shown in Fig. 2 for the case of the lateral orientation. Although not explicitly shown here, similar distributions were obtained for the anterior and oblique orientations of the NOVCoDA with respect to the proton beam incidence direction.

The FN production predominates at shallower depths, whereas the PG production is more dominant towards the end of the beam’s range in the patient. Thus, imaging the points of origins of FNs and PGs in the patient may reveal complementary information on the overall radiological path of therapeutic protons.

**Figure 3:**
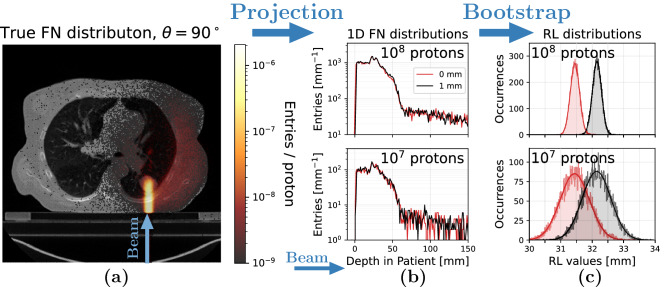
Generation of RL distributions from FN and PG production data. The figure illustrates the procedures utilized to obtain RL distributions. First, the true FN points of origin in the patient, (**a**), are mapped onto a 1D histogram along the incident proton beam direction. Examples of such histograms are shown in (**b**) for true proton beam range shifts of ${0}\,\hbox {mm}$ and ${1}\,\hbox {mm}$ at proton intensities of $10^8$ and $10^7$. From these distributions, individual RLs are calculated. Then, sub-sampling bootstrapping is used to obtain a distribution of RLs at varying proton intensities. Example RL distributions are shown in (**c**) for proton intensities of $10^8$ and $10^7$. The distributions get wider at lower proton intensities with a resulting increase in overlapping portions. Although the example illustrates the use of FN data, the procedure is the same for PG data. Note also that the procedure is similar for reconstructed FN and PG distributions with the difference being the mapping of reconstructed FN and PG production distributions in 2D onto a 1D histogram.

**Figure 4:**
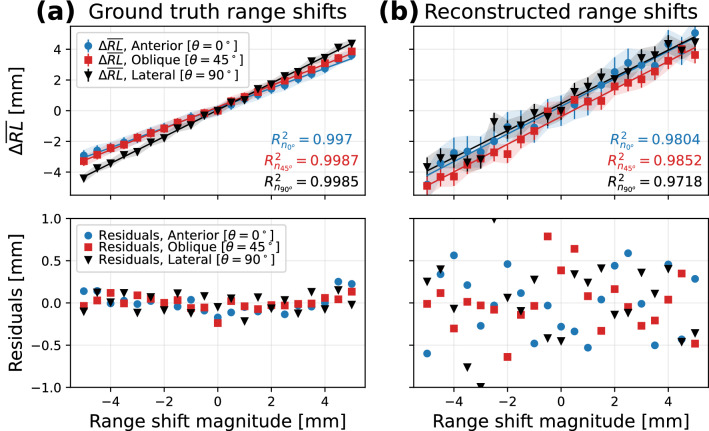
The correlation between the true range shifts and $\Delta \overline{RL}$. (**a**) Shows the true range shifts against the mean of the calculated range shifts, $\Delta \overline{RL}$, from the true distributions of detected FNs. (**b**) Shows the true range shifts against $\Delta \overline{RL}$ from the reconstructed distributions of detected FNs along with the residuals. For both (**a**) and (**b**), $\Delta \overline{RL}$ are calculated for a proton intensity of $10^{8}$ protons/spot. Use of the true distributions predicts a nearly perfect linear relationship between the true range shifts and $\Delta \overline{RL}$. The same can be said to be true for the relation between the true range shifts and $\Delta \overline{RL}$ values estimated from the reconstructed distributions in spite of increased fluctuations reflected in the lower panel showing the residuals.

For comparison to these so-called *ground-truth* distributions, the one-dimensional projected production distributions were then reconstructed from the simulated detector response using the LM-MLEM method (see “Methods” section for details). RL values were extracted from these distributions, and a sub-sampling bootstrap approach allowed the calculation of RL distributions (see Fig. 3 for an example illustration of the projection and bootstrap procedure). The weighted average of the RL distributions, $\overline{RL}$, and the subsequent difference between two such weighted averages, $\Delta \overline{RL}$, was found to be a good predictor for the range deviation, as a linear relation was observed between the true range shifts and $\Delta \overline{RL}$ for both the ground-truth and the reconstructed projections for all orientations of the NOVCoDA (see Fig. 4 for the results obtained using the FN distributions).

For both the true and the reconstructed FN production distributions, the results indicate a strong correlation between the true range shifts and the $\Delta \overline{RL}$ (see Fig. 4 for the numerical values). Similar results (i.e. $R^2$ values) were obtained for the PG distributions as well as FN and PG summed distributions (Supplementary Figs. 1 and 2).

### Minimum detectable range shifts of the system

Minimum detectable range shifts as a means of quantifying the sensitivities were obtained for all orientations of the NOVCoDA (i.e., $\theta = 0^{\circ }, 45^{\circ }$ and $90^{\circ }$) as a function of the number of protons in a single spot for FN and PG distributions individually (reconstructed through LM-MLEM) as well as for the summed distributions. The relevant proton beam intensities ranged from a minimum of $10^6$ protons/spot to a maximum of $10^8$ protons/spot, representing weak to strong proton beam spots.

**Figure 5:**
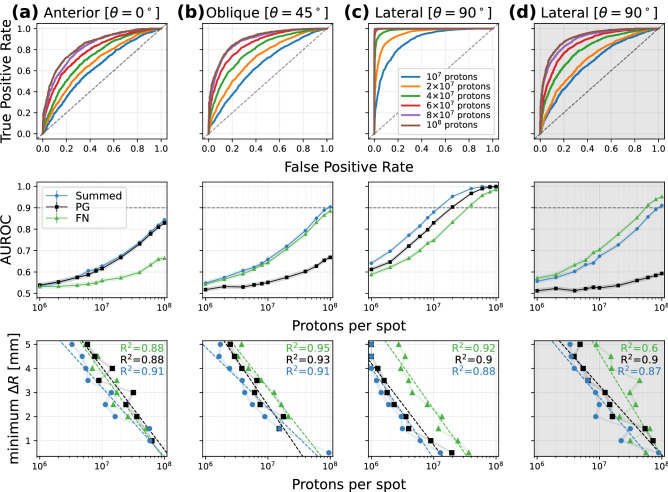
Receiver Operating Characteristic (ROC) curves, and average Area Under ROC ($\overline{{\textbf {AUROC}}}$) curves for a range shift of ${0.5}\,\hbox {mm}$ and minimum detectable range shifts. The upper panel shows the ROC curves evaluated at a range shift magnitude of ${0.5}\,\hbox {mm}$ for the reconstructed FN and PG combined data-set. The middle panel shows the calculated $\overline{AUROC}$ for the same data-set at the same range shift magnitude. The lower panel shows the minimum detectable range shifts as a function of the number of protons per spot. Note that ROCs are only plotted for proton intensities ranging from $10^7$ protons/spot to $10^8$ protons/spot whereas $\overline{AUROC}$ and minimum $\Delta R$ are plotted for proton intensities ranging from $10^6$ protons/spot to $10^8$ protons/spot. Each of these are shown for a NOVCoDA orientation of $\theta = 0^{\circ }$ (**a**), $\theta = 45^{\circ }$ (**b**) and $\theta = 90 ^{\circ }$ (**c**), as well as the $\theta = 90 ^{\circ }$ case with the estimated effects of time, energy, and position resolutions (and segmentation of a realistic detector) included in (**d**). The uncertainties in terms of one standard deviation are shown as bands around the $\overline{AUROC}$ values.

A Gaussian Naive Bayes (GNB) classifier was trained on bootstrapped projections for proton beam intensities between $$ 10^6$$ and $$10^8$$ protons/spot. The range detection sensitivity of the system was then quantified by the $$\overline{AUROC}$$ (see the “Methods” section for a definition of this parameter), with an $$\overline{AUROC} > 0.9$$ signifying a sufficient detection sensitivity. The $$\overline{AUROC}$$ obtained for the FN and PG combined distributions are given in Fig. 5 for the anterior, oblique and lateral orientations of the NOVCoDA, exemplarily for a true range shift magnitude of $${0.5}\,\hbox {mm}$$. The minimum detectable range shift, as defined by the minimum true range shift with an $$\overline{AUROC} > 0.9$$, is depicted as a function of the number of protons per spot in Fig. 5.

For the anterior orientation of the NOVCoDA, the $\overline{AUROC}$ values did not reach the threshold level of 0.9 for any of the proton beam spots considered; neither for the FN and the PG distributions individually nor for the summed distributions. It is worth noting that the PG data are obviously much more range-sensitive than the FN data in this case. In the oblique orientation, the AUROC of the sum data reaches the 0.9 threshold just at the highest proton numbers. Here, the PG data are obviously much less range-sensitive than the FN data. The outcome is significantly different when $\overline{AUROC}$ values for the lateral orientation of the NOVCoDA are considered. In this case, the threshold level at 0.9 is reached at proton intensities of (1) $3.66(11)\times 10^{7}$ protons/spot for FN data only, (2) $1.95(10)\times 10^{7}$ protons/spot for PG data only and (3) $1.28(10)\times 10^{7}$ protons/spot for the combination of FN and PG data. Although the $\overline{AUROC}$ values reported here inevitably depend on the exact method by which RL values—and thereby $\Delta \overline{RL}$ values—are calculated, they nevertheless show a tendency for improved minimum detectable range shifts (and sensitivities) for the lateral orientations of the NOVCoDA. The minimum detectable range shifts in terms of the required proton intensities evaluated at other range shift magnitudes show the same tendencies (Supplementary Figs. 3 and 4). Also, it should be noted that the required proton intensities show the expected inverse proportionality with the true range shift magnitude, i.e. higher proton intensities are needed to accurately classify cases with smaller range shifts and vice versa. Some of the pertinent numerical results are quoted here to exemplify this for the lateral orientation. At a true range shift magnitude of ${1}\,\hbox {mm}$, the threshold $\overline{AUROC} \ge 0.9$ is reached at proton intensities of $2.44(12)\times 10^{7}$ protons/spot, $8.98(32)\times 10^{6}$ protons/spot and $6.08(21)\times 10^{6}$ protons/spot for FN data only, PG data only and the combination of FN and PG data, respectively. For the same orientation, but for a true range shift magnitude of ${2}\,\hbox {mm}$, the required proton intensities drop to $1.36(10)\times 10^{7}$ protons/spot, $3.58(10)\times 10^{6}$ protons/spot and $3.02(12)\times 10^{6}$ protons/spot for FN data only, PG data only and the sum of FN and PG data, respectively.

The presented results are clearly in favor of the lateral orientation and a combination of the information obtained by both PG and FN data. Therefore, for the more realistic case in which the true MC hit data are smeared taking into account detector resolution (time, energy, depth of interaction and segmentation), the corresponding calculations were performed only for the lateral orientation. The results are shown in Fig. 5d and we see that the $\overline{AUROC}$ values calculated using the PG data never reach the threshold of 0.9. For the FN data only, the threshold is reached at proton intensity of $5.57(14)\times 10^{7}$. For the summed FN and PG data, the threshold is reached at proton intensity of $8.86(47)\times 10^{7}$, indicating that the poorer quality of PG data negatively impacts performance. Finally, for the lateral orientation, using deposited energy cut-offs of 100 keV for incident FNs and 10 keV for incident PGs, the double and triple scatter detection efficiencies were estimated to be $2.33 \times 10^{-4}$ neutrons/proton and $2.67 \times 10^{-4}$ gamma-rays/proton for FNs and PGs, respectively. For both cases, the statistical uncertainties are less than $1 \%$ of the mean values.

### An example of a treatment deviation caused by anatomical changes

The 4D planning CT of a patient with locally advanced non-small cell lung cancer was used to determine parameters of the incident proton pencil beam such that the beam stopped inside the the target volume. This example case was distinguished by a baseline shift, i.e. a change in the patient’s breathing motion pattern causing a shift and deformation of the tissue and lymph node metastasis inside the target volume, that was captured on the repeat 4D CT scan performed during the first treatment week.

**Figure 6:**
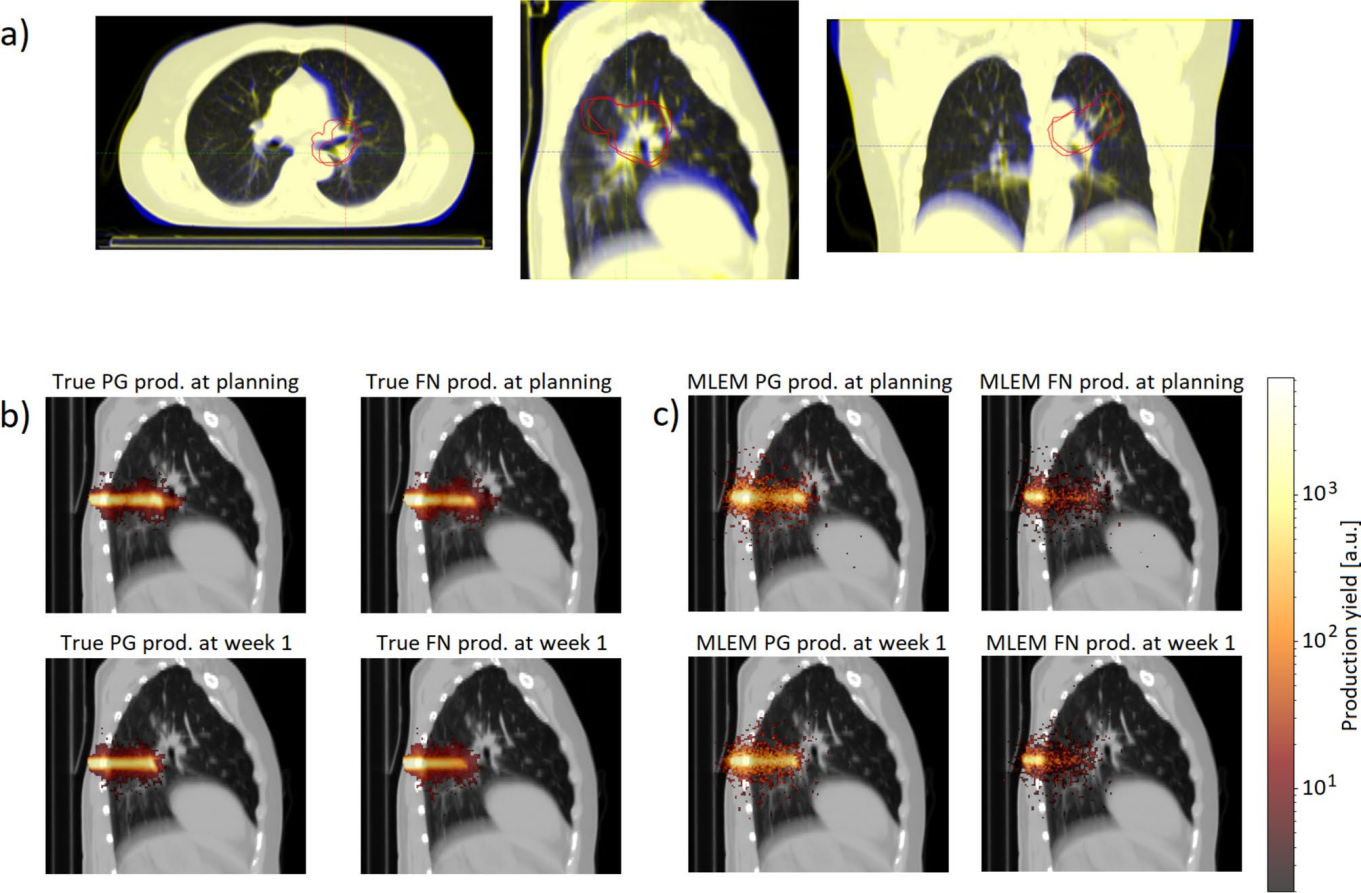
Example of treatment deviation caused by anatomical changes. (**a**) The axial, sagittal and coronal plane of the example patient with the average intensity projection of the 4D planning CT (yellow) overlaid on the repeat CT (blue). The target volume contoured by the treating oncologist on both CTs are visible as red contours. Both a shift and deformation of the target volume with corresponding change in tissue densities can be observed. The lungs are larger on the planning compared to the repeat CT indicating a shallower breathing pattern during acquisition of the latter. (**b**) PG and FN production, both from MC truth and reconstructed with MLEM, from the planning CT (top) and when produced in the week 1 CT, overlaid on the planning CT (bottom).

**Figure 7:**
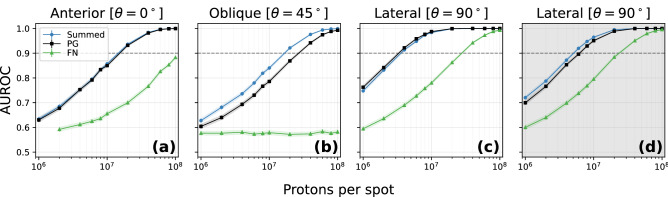
$\overline{\textit{AUROC}}$ curves for the clinical case at three different orientations of the NOVCoDA with respect to direction of the incident proton beam. The figure summarizes the calculated $\overline{AUROC}$ values using only FN and only PG distributions reconstructed using the LM-MLEM algorithm as well as for the combination of FN and PG distributions for an orientation of $\theta = 0 ^{\circ }$ (**a**), $\theta = 45 ^{\circ }$ (**b**) and $\theta = 90 ^{\circ }$ (**c**), as well as the $\theta = 90 ^{\circ }$ case with the estimated effects of time, energy, and position resolutions (and segmentation of a realistic detector) included in (**d**). Statistical uncertainties in terms of one standard deviation of the $\overline{AUROC}$ are also shown explicitly as a narrow band around each curve.

It should be noted that, in this case, there is no explicit information on the magnitude of the true range shift caused by this baseline shift; however, there is a distortion in the delivered dose from the primary pencil beam due to a change in the position of the tumor and, therefore, a change in the overall production distributions of both PGs and FNs (see Fig. 6). The observed baseline shift could be due to changes in the breathing patterns of the patient as well as other unknown factors. As such, no attempt was made to determine the $\Delta \overline{RL}$ values from reconstructed (or ground truth) FN and PG production distributions. Rather, the focus is on using the RL distributions generated through bootstrapping of the FN and PG production distributions from the **planning CT** and the **one week CT** data-sets in conjunction with the GNB classifier. Upon training the GNB classifier and repeated calculations, the discrimination capability of the NOVCoDA in terms of the $\overline{AUROC}$ values was estimated. In order for the discrimination capability to be considered to be at an adequate level, the same threshold level, i.e. $\overline{AUROC} \ge 0.9$, had to be reached. This was done using both the LM-MLEM reconstructed and ground truth production distributions of FNs and PGs (and the sum of FN and PG data) for all three orientations of the NOVCoDA. The $\overline{AUROC}$ values estimated from the LM-MLEM reconstructed distributions are summarized in Fig. 7 for proton intensities ranging from $10^6$ to $10^8$. See supplementary Fig. 5 for the corresponding values calculated for the ground-truth distributions.

Also for the clinical example considered here, the lateral ($\theta = 90 ^{\circ }$) orientation of the NOVCoDA is clearly favored over the anterior ($\theta = 0 ^{\circ }$) and oblique ($\theta = 45 ^{\circ }$) orientations, with information extracted from PG distributions outperforming that of FN distributions for all three orientations. For example, for the anterior orientation, the required proton intensities to correctly identify the baseline shift are at $1.61(10)\times 10^{7}$ protons/spot and $1.54(10)\times 10^{7}$ protons/spot for PG and summed distributions, respectively. The corresponding $\overline{AUROC}$ values for the FN distributions do not reach the threshold of 0.9 at any of the considered proton intensities. Similar trends are observed for the oblique orientation of the NOVCoDA. In addition, for the same orientation, the $\overline{AUROC}$ values estimated for FN data only do not follow the increasing trend as the proton intensity is increased. This is likely attributed to imaging artifacts as the trend is clearly increasing when ground-truth FN distributions are instead utilized. For the lateral orientation, spatial information extracted from the distributions of both particle species and their sum show a significant improvement with the required proton intensities of $2.94(11)\times 10^{7}$ protons/spot, $3.46(11)\times 10^{6}$ protons/spot and $3.67(11)\times 10^{6}$ protons/spot for the FN, PG and summed distributions, respectively. For the lateral orientation, the discrimination capability of the NOVCoDA was also studied taking into account detector resolution. It turns out that the NOVCoDA exhibits useful discrimination capabilities even when detector resolution is factored in. The minimum required proton intensities to identify the baseline shift are at $2.47(13)\times 10^{7}$ protons/spot, $6.24(25)\times 10^{6}$ protons/spot and $5.18(18)\times 10^{6}$ protons/spot for FN, PG and the summed distributions, respectively.

## Discussion

PT holds great potential to reduce integral dose to healthy tissue in patients undergoing RT for cancer treatment and thereby reduce the treatment toxicity. However, range uncertainties that may be caused by anatomical changes, setup errors, organ motion as well as uncertainties in estimations of the tissue stopping power pose a significant challenge in routine clinical practice and prevent benefiting from the full tissue-sparing potential of PT. Range uncertainties require the development of new imaging and radiation detection devices to enable real-time range monitoring during treatment. In spite of significant international efforts, reliable real-time range monitoring still remains an unresolved problem in PT. In this study, we proposed the use of a novel imaging device, so-called NOVCoDA, with the capability of detecting and imaging points of origins of secondary FNs and PGs produced in nuclear interactions in tissue during treatment. The NOVCoDA will take advantage of the highly penetrating secondary FN and PG emissions in PT and be able to discriminate interactions induced in its sensitive volume by these two particle species through employing novel organic scintillation materials with enhanced pulse shape discrimination capabilities.

The main focus of the study at hand was to explore, through MC simulations of mono-energetic proton pencil beams with therapeutic energies and intensities impinging on realistic patient models, the feasibility of the proposed NOVCoDA and, in general, the yet unexplored concept of dual FN and PG imaging for correct identification of range deviations in the context of PT, also including detector resolution effects. A methodological novelty of the study at hand was the use of such CT-scans of patient models in conjunction with the MC simulations that include realistic tissue heterogeneities that pose challenges to any imaging device designed for the purposes of range monitoring in PT but are often ignored in other studies. Another important aspect of the study was the use of realistic reconstruction of the production distributions of FNs and PGs through detailed simulations of the interactions of FNs and PGs in the sensitive volume of the NOVCoDA and the subsequent use of the LM-MLEM algorithm for image reconstruction from intersections of kinematically constructed FN and PG event cones with planes coinciding with the primary proton beam axis. For the FN and PG distribution reconstructions where detector resolution effects were taken into account, LM-MLEM algorithm was not used as the current implementation does not include any suitable regularization techniques. On the other hand, a reconstruction based solely on cone-plane intersections is much faster than the iterative LM-MLEM algorithm.

The sensitivity of the NOVCoDA to detect range shifts at various proton intensities was quantified through a controlled erosion and dilation of the outer layers of the patient model. Firstly, the sensitivities were studied using the NOVCoDA data sets that did not include detector resolution effects for three different orientations of NOVCoDA, i.e. anterior, oblique and lateral with respect to the incident proton beam direction. Using the true collision parameters, calculating a simple RL parameter from the resulting FN and PG distributions (and their linear sum), as well as a bootstrapping procedure applied to enable statistical analyses of the resulting RL distributions, show that the NOVCoDA provides the highest sensitivity at the lateral orientations ($\theta = 90^{\circ }$) with respect to the incident proton beam direction. It is also noteworthy that the sensitivity is generally improved when the PG and FN distributions are summed and used simultaneously. In addition, simulations of an example lung cancer patient with a baseline shift representing an anatomical change revealed similar tendencies. Secondly, as lateral orientations are clearly favored in terms of the range shift detection sensitivities and identification of clinically relevant treatment deviations, the sensitivities were quantified, only for the lateral orientation ($\theta = 90^{\circ }$), also for a more realistic case where the true collision parameters are smeared using experimentally determined energy (see the “Methods” section for the parametric description of the energy resolution and the parameter values), time (${500}\,\hbox {ps}$) and depth-of-interaction (${11.775}\,\hbox {mm}$) resolutions, incorporating also segmentation effects via re-sampling of the remaining two collision coordinates from uniform distributions of width ${5}\,\hbox {mm}$. The resolution data are obtained in a series of experimental characterization campaigns of one of the most suitable scintillator materials, i.e., OGS, of relevant form factors, performed at the nELBE neutron time-of-flight facilities in Dresden, Germany. The details of these campaigns are to be published elsewhere. When detector resolution is accounted for, the results of the current study show that the NOVCoDA could feasibly reveal range shifts of around ${1}\,\hbox {mm}$ at clinically relevant proton intensities ($22.30(13)\times 10^{7}$ protons/spot) as FN and PG data were summed to yield a composite distribution. Moreover, simulations of an example lung cancer patient with a baseline shift resulting in a deviation from the planned treatment showed the feasibility of the NOVCoDA to detect realistic treatment deviations at clinically relevant proton intensities ($5.18(18)\times 10^{6}$ protons/spot) when using linear sum of reconstructed FN and PG distributions. In general, the overall results of this study favor the lateral ($\theta = 90^{\circ }$) orientations of the NOVCoDA and the summation of FN and PG distributions. It should also be mentioned that, in the example anatomical change considered in this work, the information extracted from PG distributions seems to be superior to that of FN distributions when it comes to detection of the proton beam range shift caused by that specific anatomical change (see Fig. 7d). In this regard, it is important to point out that this will also depend on the nature of the deviation that causes a given range shift. In this specific example, the baseline shift leads to an anatomical change near the end of the primary beam range where the PG production predominates. This, in combination with the fact that the FNs are mostly produced in the forward direction, i.e. in the direction of the primary beam, might partially explain the poorer performance of spatial information extracted from the FN distributions. If changes occurring at shallower depths are the cause of an undesired range shift, then the overall spatial information extracted from FN distributions may have improved discriminatory capabilities.

Some limitations of the study at hand must be mentioned. An important limitation was the fact that energy-dependent depth-of-interaction resolution is not yet accounted for. This will be implemented in future modelling work that will accompany experimental investigations of the NOVCoDA in laboratory experiments as well as validation efforts in medical proton beamlines. Detailed experimental campaigns are underway where also pulse shape discrimination capabilities of relevant scintillator materials such as the OGS are being quantified. The results of these experiments will be published elsewhere. Although we have, in this feasibility study, accounted for lower-energy detection limits of ${100}\,\hbox {keV}$ for FNs and ${10}\,\hbox {keV}$ for PGs, the practical detection limits will, to a large extent, depend on the final readout architecture as using photodetectors with intrinsically high gain (gain factors of $10^6$–$10^7$) will allow detection of events with low deposited energies. The detection limits will also be affected by the quality of the photodetector-scintillator coupling as well as the overall quality of the surface finish of the scintillators. Concerning limitations on image reconstruction, LM-MLEM was only used on noise-free data obtained directly from true MC simulated collision parameters. If the LM-MLEM or similar iterative techniques are to be applied in conjunction with the collision data that also incorporate the deteriorating detector resolution effects, appropriate regularization techniques will need to be carefully considered. Another important aspect is the method by which RLs are calculated is likely to have an effect on the final range-monitoring accuracy. Therefore, a multivariate statistical modelling study has also been initiated to determine the RL calculation method or combinations of methods whereby the accuracy can be improved^24^.

Despite the above-mentioned limitations, the results of the study have some important implications for a future adaptation of the NOVCoDA for range-monitoring purposes. Human tissue is also rich in efficient neutron scattering nuclei, and one issue that could be raised is the fact that FNs would scatter and thereby lose the information on their points of origin prior to being detected in the NOVCoDA, thereby diminishing the capabilities of the NOVCoDA to monitor range shifts based on imaging of incident FNs. The results presented in this work show that scatter in the patient is not expected to be an issue as the full three dimensional patient geometry and material composition are already accounted for in the MC models. Also, the results seem to imply that the background signal in the PG component due to neutron activation in the surroundings or human tissue will not have a significant influence on the accuracy as MC simulated PG production included all PG production, including those from neutron activation via inelastic fast neutron scatter or thermal neutron capture reactions. Another important implication of the results is the fact that strong correlations between range shifts calculated from spatial information extracted from reconstructed FN and PG distributions and true range shifts do not necessarily guarantee a good response. Although coefficients of determination for all three orientations of the NOVCoDA all indicate a strong correlation to the true range shifts (see Fig. 4), the minimum range shift detection limits are influenced by the orientation of the NOVCoDA imaging system with respect to the incident proton beam direction (see Fig. 5), with the lateral orientation ($\theta = 90 ^{\circ }$) being clearly favored over the anterior ($\theta = 0^{\circ }$) and oblique ($\theta = 45^{\circ }$) orientations. This can be likely attributed to imaging artifacts that appear for the anterior and oblique orientations of the NOVCoDA, but not for the lateral orientation (see supplementary Fig. 6). Imaging artifacts of a similar nature, appearing as a blurring artifact, are also encountered in Compton imaging systems and are attributed to data incompleteness (projection loss due to the finite detector size)^25^. Thus, for a future clinical application of the NOVCoDA, it would be important to focus research efforts also on algorithmic and theoretical improvements in image reconstruction techniques that can minimize the unwanted effects of such artifacts. In this regard, fully Bayesian approaches to image reconstruction might be advantageous over the more conventional MLEM, and will be considered as part of future work.

The sensitivity of PT to treatment deviations and the presence of such deviations during treatment will always be a limiting factor towards the full exploitation of its dosimetric advantages over the more conventional radiotherapy. To this end, online adaptive PT is gaining more traction where dose plans can be adapted in each treatment fraction to limit the dose to healthy tissue and ensure that the prescribed dose is given to the tumorous tissue^26^. The NOVCoDA will provide in-vivo monitoring and verification of the delivered dose (or at least the particle beam range in tissue) which is a vital component of such systems providing real-time or near real-time information on the dose delivery to decision-support systems. Although a number of state-of-the-art range-monitoring systems are now being tested in clinical settings, their relatively large clinical footprint limit their applications to certain treatment sites. The NOVCoDA with its reduced clinical footprint (in terms of both size and weight; the total scintillator volume considered in this work, i.e. $30 \times 20 \times 20$ $\hbox {cm}^{3}$, corresponds to a total sensitive material mass of only about ${12}\,\hbox {kg}$.) in relation to these systems, will enable range monitoring in different clinical scenarios and for different treatment sites. More importantly, NOVCoDA will, on the contrary to the state-of-the-art systems that treat the neutron component as background signal degrading performance, enable the use of this radiation species, that is also produced in abundance and is an unavoidable byproduct of the treatment, for range-monitoring purposes.

A brief literature search justifies the clinical relevance of the NOVCoDA and the multi-particle imaging concept outlined in this work; Polf et al.^19^ states that range monitoring systems intended for clinical applications in standard PT must provide a good performance for single spots of $10^7$ to $10^8$ protons/spot. The results presented in this work, especially those that also take into account detector resolution effects, clearly indicate that the NOVCoDA has the potential to provide range shift detection capabilities at the expected levels for clinically relevant proton beam intensities, the caveat being a seeming dependency on the orientation of the NOVCoDA-imager with respect to the incident beam direction. Also, in light of previous results reported in other simulation studies, such as the one by Smeets et al.^8^, where range shift detection sensitivities of ${1}\,\hbox {mm}$ have been achieved down to intensities of $5 \times 10^8$ protons/spot, it can be stated that the results of this work show that the NOVCoDA could be a feasible approach to range monitoring in PT.

Finally, although the focus of this manuscript has been on proton therapy scenarios, it is a physics fact that FNs and PGs are also produced abundantly in other PT modalities such as He- and C-ion therapy^22,27,28^ where the same clinical challenges related to range uncertainties are also present. Therefore, it is expected that the use cases of the NOVCoDA can also be extended to range-monitoring applications within these therapy modalities to further reduce treatment toxicities.

## Methods

### Image reconstruction from FN and PG cones

The image reconstruction relies on the kinematic reconstruction of FN and PG event cones that describe the possible incidence directions of individual FNs and PGs, confined to the surface of the event cones. The kinematic reconstruction of FN and PG cones in the NOVCoDA relies, in turn, on the detection of individual FN and PG collisions in the sensitive volume of the NOVCoDA as illustrated in Fig. 1D.

For FNs, the non-relativistic kinematic reconstruction of event cones relies on two successive elastic scatters on hydrogen nuclei, i.e. (n,p) scatters. In the first scatter, the incident neutron transfers part of its kinetic energy to the recoiling proton. The transferred energy depends on the scattering angle. The scattered neutron will have its energy reduced. The scattered neutron then needs to undergo another (n,p) scatter in the sensitive volume of the NOVCoDA. The second (n,p) collision is used to determine the FN *time-of-flight*, $\tau$, between the first and second scatters. The energy of the scattered neutron can then be calculated as:

1 $$\begin{aligned} E_n^{'} = \frac{1}{2}m_{n}\bigg (\frac{d}{\tau }\bigg )^2 \end{aligned}$$

where $E_n^{'}$ is the kinetic energy of the scattered neutron, $m_n$ is the mass of neutron, $\tau$ is the time-of-flight, and *d* is the distance between the first and second collisions sites (whose positions are experimentally determined by comparing the difference in signal strength and/or arrival time in the two ends of each bar struck within the NOVCoDA). Then, a best estimate of the incident FN energy can be calculated as $E_n = E_n^{'}+E_p$ where $E_p$ is the kinetic energy of the recoiling proton in the first (n,p) scatter (experimentally determined from scintillator light response). Once the energy of the incident FN is estimated, the polar scattering angle, $\theta$, in the laboratory-frame can be estimated as:

2 $$\begin{aligned} \theta = \sin ^{-1}{\sqrt{\frac{E_p}{E_n}}} \end{aligned}$$

The azimuthal angle, $\phi$, remains unresolved, thus $\phi$ : [0,2$\pi$]. This leads to formation of an event cone with its apex at the first (n,p) scatter coordinates in $\mathrm{I\!R^3}$ and direction along the vector joining the first and second (n,p) scatter coordinates. In this work, the use of non-relativistic collision kinematics is justified as $\beta = v/c < 0.1$ on average (for scattered neutrons), however, for higher energy neutrons, relativistic versions of the kinematic equations should be used.

For PGs, the corresponding reconstruction of event cones relies on triple coincident collisions, mostly in the form of Compton scatter, in the sensitive volume of the NOVCoDA. This is because the incident PGs will typically have high energies and because the NOVCoDA consists of low density, low atomic number materials, meaning that photoelectric absorption is less likely to occur. Using Compton collision kinematics, one can arrive at:

3 $$\begin{aligned} E_\gamma = \Delta {E_1} + 0.5 \bigg [\Delta {E_2} + \sqrt{\Delta {E_2}{^2} + \frac{4\Delta {E_2}m_{e}c^2}{(1-\cos \theta _2)}}\bigg ] \end{aligned}$$

where $E_\gamma$ is the energy of the incident PG, $m_{e}$ is the electron rest mass, *c* is the speed of light, $\theta _2$ is the scattering angle in the second collision, and $\Delta {E_1}$ and $\Delta {E_2}$ are the energies deposited in the first and second collisions, respectively. Once the energy of the incident PG is known, the scattering angle $\theta _{1}$ (in the first collision) can be calculated from the Compton scatter kinematics as:

4 $$\begin{aligned} \cos \theta _1 = 1 + m_{e}c^2 \bigg [\frac{1}{E_\gamma } - \frac{1}{E_\gamma ^{'}} \bigg ] \end{aligned}$$

where $E_\gamma ^{'}$ is the energy of the scattered PG following the first scatter event in the NOVCoDA. The azimuthal angle remains, as in the case of FNs, unresolved with these measurements and therefore, $\phi$ : [0,2$\pi$]. The apex of the PG cone is then located at the spatial coordinates of the first Compton scatter event in $\mathrm{I\!R^3}$. The PG cone then has a direction along the vector joining the first and second collision sites.

For reconstructing images of PG and FN distributions in the patient, each event cone was discretized into *n* (*n* = 1000 in this work) equi-spaced rays (see Fig. 1D). Each ray was then traced onto a plane containing the proton beam. We then find geometric ray-plane intersections, i.e. points in $\mathrm{I\!R^2}$. Next, we find the pixel in an $N \times N$ image that contains the current point and increment the corresponding pixel counts by 1. It is important to note that pixel counts can be incremented only once by a given cone, meaning multiple rays in a given pixel lead to an increment by only 1. Once all kinematically reconstructed event cones are processed in this way, an iterative List-Mode Maximum Likelihood Expectation Maximization (LM-MLEM) algorithm^29^ was executed, for a pre-determined number of iterations, to obtain the final reconstructed PG and FN distributions in $\mathrm{I\!R^2}$. At this stage, no smoothing penalties were included in the implementation. The update equation for the LM-MLEM algorithm is given as:

5 $$\begin{aligned} \lambda _j^{n+1} = \frac{\lambda _j^{n}}{s_j} \sum _{i\in j}{\frac{t_{ij}}{\sum _{j^{'} \in i} {t_{ij^{'}}\lambda ^n_{j^{'}}}}} \end{aligned}$$

where the first sum is over events, *i*, for a given pixel *j*; the second sum is over pixels, $j^{'}$, for a given event *i*; $\lambda _j^{n+1}$ is the $j{\mathrm {th}}$ pixel value in iteration $n+1$; $\lambda _j^{n}$ is the value of the $j{\mathrm {th}}$ pixel in iteration *n*; $s_j$ is the sensitivity of the $j{\mathrm {th}}$ pixel; and $t_{ij}$ are the elements of the system matrix, which is a large sparse matrix (in LM-MLEM, each event, *i*, represents a single event cone which intersects only a small fraction of the $N \times N$ image pixels) constructed by event-by-event processing of the event cones as described above.

As a means of regularization, the LM-MLEM execution was stopped at 100 iterations. This ensured convergence of the average pixel densities without introducing noise pick-up and subsequent divergence. For simplicity, the sensitivities of each pixel in the image space was set to 1.0, which is a reasonable approximation^29^, although it does not account for self-attenuation or non-uniform detection efficiencies.

Furthermore, the true Monte Carlo (MC) collision data are smeared using realistic resolution characteristics of organic glass scintillator (OGS) samples. The true detection time of each event is smeared using a coincident time resolution (FWHM) of ${500}\,\hbox {ps}$, the position of each event is smeared using depth of interaction resolution of ${11.775}\,\hbox {mm}$, and re-sampled from a uniform distribution with a width of ${5}\,\hbox {mm}$ to account for segmentation effects assuming a segment size of 10 $\times$ ${10}\,\hbox {mm}^{2}$, and the energy deposited in each event is smeared using a parametric description of the energy resolution given as:

6 $$\begin{aligned} R(L) = \sqrt{\alpha ^2 + \frac{\beta ^2}{L} + \frac{\gamma ^2}{L^2}}, \end{aligned}$$

where $\alpha = 0.0497 \pm 0.0031$, $\beta = 0.0000 \pm 8.4221$ $MeVee^{1/2}$ and $\gamma = 0.0349 \pm 0.0011$ $MeVee^{2}$. These parameters have been determined through a curve fit to a limited number of resolution values measured experimentally for an OGS sample of 10 $\times$ 10 $\times$ ${100}\,\hbox {mm}^{3}$. This is also the major reason for the large uncertainties in especially the $\beta$ parameter. The details of the experimental campaigns are to be published elsewhere. For PGs, the calculated resolution values are used as is whereas for FNs, the values are scaled by a factor of two to partially account for the light quenching effects. Also, prior to smearing the true MC data, a particle-specific deposited energy cut is applied, i.e., ${10}\,\hbox {keV}$ for PG induced events and ${100}\,\hbox {keV}$ for FN induced events. The data sets with the deposited energy cuts have also been used to estimate the FN and PG detection efficiencies. It should be further pointed out that, for the data sets that also account for detector resolution effects, the image reconstruction is only performed at the level of geometric ray-plane intersections due to the fact that our implementation of the LM-MLEM algorithm does not include any suitable numerical regularization techniques.

### Range landmark and range shift determination

We determine so-called range landmarks (RLs) by taking one-dimensional projections of both the reconstructed and Monte Carlo (MC) ground truth distributions of PGs and FNs as well as projections of the summed distributions (see Fig. 1E). The projection is along the depth direction, i.e. along the direction of the primary proton beam. For ground truth distributions, the depth coordinates of the ensemble of PGs and FNs are binned using ${1}\,\hbox {mm}$ bin widths. The reconstructed data are readily binned into ${2}\,\hbox {mm}$ bins prior to execution of the LM-MLEM algorithm, and, thus, no additional binning is needed. The weighted average of the binned data are then calculated as follows: $\overline{x} = \frac{\sum _{i=1} ^{n} w_i x_i}{\sum _{i=1} ^{n} w_i}, w_i = \frac{x_i}{\sum _{i=1} ^{n} x_i}$ where $x_i$ are the $i\mathrm{th}$ bin counts and $w_i$ are the weights of the $i\mathrm{th}$ bin. The weights are normalized such that $\sum _{i=1} ^{n} w_i = 1$. The RL for each distribution was then set equal to the weighted average of the binned data. This method is used in conjunction with a bootstrapping approach to determine the average RL, $\overline{RL}$, values for each case.

To determine $\overline{RL}$ and the statistical uncertainties associated with each $\overline{RL}$ calculation, we employed a bootstrapping approach with replacement^30^. The sub-sampling bootstrapping algorithm, as implemented in this work, is described as follows:

1. Determine bootstrap sample size, *N*, from the ratio of proton intensities. If $\sigma _{\overline{RL}}$ is to be determined at a proton intensity of $10^{8}$, then the sample size, *N*, is calculated as: $N = \frac{N_{\mathrm {total}}}{N_{\mathrm {histories}}/10^{8}}$ where $N_{total}$ is the total number of valid events in a given MC run and $N_{histories}$ is the total number of simulated protons.
2. Depending on whether the dataset to be analyzed is the MC ground truth distributions or the reconstructed distributions, the following steps were implemented:
  1. *For the ground truth distributions:* From the entire data set, randomly sample coordinate values to fill an array of size *N* with replacement, meaning that the same data points from the main data set can appear multiple times in the sampled array.
  2. *For the reconstructed distributions:* Reconstruct the FN and PG distributions with LM-MLEM for the entire dataset once and construct a one-dimensional profile by taking a slice through the reconstructed 2D images that coincides with the proton beam axis. Re-scale the bin values as described in step 1. Re-sample each bin value from a *Poisson* distribution with a mean, $\mu$, set to the re-scaled and re-sampled bin value. Repeat this for each bin in the one-dimensional histogram.
3. Calculate the RL for the data set sampled in step 2 by calculating the weighted average of the one-dimensional histogram.
4. Repeat steps 2 and 3 for a fixed number of iterations to generate a distribution of calculated RL values. In this work, we set the number of bootstrap iterations to $10^{4}$ to minimize the statistical variation in the RL uncertainty estimates. This step also represents the inner loop of the algorithm.
5. Go back to step 1, and re-determine the bootstrap sample size, *N*, for a different proton intensity and repeat steps 2, 3 and 4 to get the RL uncertainty at varying proton intensities.

It is important to point out that the method described herein predicts that RLs, in line with the Central Limit Theorem, follow a Gaussian distribution, $\mathcal {N}\left( \overline{RL},\,\sigma _{\overline{RL}}^{2}\right)$. Thus, the $\overline{RL}$ uncertainty is taken as the standard deviation of the resulting normal distributions.

The range shift determination relies on the calculation of RLs as explained above. The calculated $\overline{RL}$ show a linear dependence on the true range of the proton beam. Therefore, a shift in the true range of the proton beam will result in a corresponding shift in the position of the $\overline{RL}$. The correlation between the shifts observed in $\overline{RL}$, i.e. $\Delta \overline{RL}$, and actual range shifts as implemented in the MC models was quantified using the coefficient of determination, $R^2$.

### Range shift limit determination

A probabilistic classification of bootstrapped RL values was performed through a binary Gaussian Naive Bayes classifier (GNB)^31^. The implementation was done using python’s *scikit-learn* library^32^. The calculated RL values for a given particle species or their combination were labelled as belonging to a class corresponding to “no range shift” or “range shift” as the true labels of each RL value. The bootstrapped “no range shift” RL values were obtained using the original dataset without any dilation or erosion, and the corresponding “range shift” RL values were obtained using the dilated and eroded datasets at different proton intensities. The labelled RL values were then split into a training (80%) and a test (20%) set, chosen randomly. To avoid potential bias due to unbalanced datasets, the training of the classifier is done individually in each case. This is also supported by the fact that we are interested in determining the classification performance with respect to the original dataset at different proton intensities. As we only have one feature per class, the likelihood of each RL value to belong to one of the two classes is given as:

7 $$\begin{aligned} p(RL|c_j) = \frac{1}{\sqrt{2\pi \sigma ^2_j}} \exp \bigg [ {-\frac{1}{2} \bigg ( \frac{RL-\overline{RL}_j}{\sigma _j} \bigg ) }^2 \bigg ] \quad \text {for j=1, 2} \end{aligned}$$

Then, the MAP (maximum a posteriori) estimate of the posterior probability and the most likely class (“no range shift” versus “range shift”) to which a new observation of RL values can be assigned is given as:

8 $$\begin{aligned} C_j = \underset{c_j}{\arg \max }\ p(c_j|RL) \quad \text {for j=1, 2} \end{aligned}$$

The NOVCoDA’s range shift detection limit is then determined based on the accuracy of the binary GNB. The training and test sets, both of which contain bootstrapped RL values, are used to train the classifier for different cases accounting for FN and PG particle species individually and their combination at different proton beam intensities ranging from $10^6$ to $10^8$ protons. As such, the statistical uncertainties in the calculated RL values are implicitly accounted for. For each range shift case, the GNB classifier was re-trained and re-tested on the same data set for a preset number of iterations ($n=1000$), with a randomized selection of training and test sets for each trial. This allowed for calculations of an average *Area Under the Receiver Operating Characteristic Curve* ($\overline{AUROC}$)^33^. The detection limit in terms of the required proton intensity was then decided based on a threshold of $\overline{AUROC} \ge 0.9$. $\overline{AUROC}$ values were linearly interpolated to obtain a more accurate estimation of the required proton intensities. Repeated evaluations of AUROC as described here also allowed estimations of statistical uncertainties of the $\overline{AUROC}$. These were then used to estimate the corresponding uncertainties in the range shift detection limits in terms of the required proton intensities.

### Monte Carlo simulation environment

The public patient dataset LCTSC-Test-S1-203 from “Lung CT Segmentation Challenge”^34,35^ was retrieved from the Cancer Imaging Archive^36^. The dataset contains hundreds of cancer patients; however, a single patient was used and modified for the purposes of this study. A central spot in a ${3}\,\hbox {cm}$ tumor in the left lung was chosen as the target point for a proton pencil beam.

Twenty-one range-shifted scenarios were made from this single scan using the Medical Interactive Creative Environment (MICE) Toolkit v 1.1.3 (Nonpi Medical, Umeå, Sweden): The patient body was threshold-masked, and subsequently the mask was eroded/dilated in ${1}\,\hbox {mm}$ steps up to ${\pm 5}\hbox {mm}$, depending on the direction of the range shift. The eroded area was substituted with air, and the additional area from the mask dilation was filled with water. See supplementary materials for an animation of the erosion/dilation processing of the patient model. For the intermediary ${0.5}\,\hbox {mm}$ steps, due to the ${1}\,\hbox {mm}$ voxel size, the proton beam energy was reduced by ${405}\,\hbox {keV}$.

The model simulations were performed using Geant4 10.5^37^ together with GATE v. 8.2^38^, configured with the QGSP_BIC_EMY physics building list as suggested by Wrońska et al.^39^ (without the high-precision transport of neutrons below ${20}\,\hbox {MeV}$). To reduce the simulation time, the production threshold was set to ${1}\,\hbox {cm}$ for protons and gamma rays, and to ${1}\,\hbox {m}$ for electrons and positrons. The source code of GATE was modified to include the target element of inelastic interactions in the CT-scan in the ROOT output, thus facilitating subsequent spectral analysis.

The simulations were performed in two steps. First, an ${85}\,\hbox {MeV}$ posterior-anterior proton beam with ${4.7}\,\hbox {mm}$ FWHM and ${2.5}\,\hbox {mrad}$ divergence was directed towards the center of the tumor. During the irradiation all generated neutrons and prompt gamma rays were stored in phase-space files. Second, the phase-space files were used as beam sources for the detector simulations: the output was the source position and direction of the source particles hitting the detector, as well as information about interactions inside the detector. Finally, only the phase-space information of secondaries interacting in the NOVCoDA was kept.

### An example of treatment deviations caused by anatomical changes

A clinically relevant case with locally advanced non-small-cell lung cancer patient from Haukeland University Hospital (approved by the regional committee for medical and health research ethics in Western Norway, protocol code 2019/749) was studied. Informed written consent was obtained from the patient and research was performed in accordance with the Norwegian Health Research Act and the Declaration of Helsinki. A 10-phase 4DCT was aquired for radiotherapy planning and during the first radiotherapy treatment week for this patient. Imaging was performed on a Big Bore CT scanner (Philips Healthcare, Best, The Netherlands), using a Posirest-2 support device (Civco Radiotherapy, Coralville, USA) for fixation in the supine position with arms resting above the head. The breathing curve for the 4DCT was acquired using the Philips Bellows device. For the treatment simulations we used the average intensity projections of the 4DCTs generated in Eclipse (Varian Medical Systems, Palo Alto, USA).

A central point in the delineated gross tumor volume (GTV) was chosen as the target, and the two time points were registered to that point accordingly. A single pencil beam with sufficient energy to stop in the center of the GTV, i.e. ${75}\,\hbox {MeV}$, was simulated in GATE.

The ability to separate the FN/PG production using the RL analysis analogously to the range-shift study was then evaluated.

It should be pointed out that for both the clinical example described here and the data set from the “Lung CT Segmentation Challenge”, the gantry angle was at $180^{\circ }$ such that the proton beam is going through the patient couch. This choice of gantry angle is indeed encountered in clinical cases as also shown in Yan et al.^40^.

## Supplementary Information

Supplementary Figures.

Supplementary Figure 7.

## Data Availability

The CT datasets, the Monte Carlo simulation scripts and the raw Monte Carlo generated data files used in this work are available from the corresponding authors upon reasonable request. The CT data of the clinical case are not publicly available due to privacy reasons as they are part of an ongoing study.

## Abbreviations

PT: Particle therapy
RT: Radiotherapy
PG: Prompt gamma ray
FN: Fast neutron
NOVCoDA: The NOVO compact detector array
OGS: Organic glass scintillator
MC: Monte Carlo
RL: Range landmark
MLEM: Maximum Likelihood Expectation Maximization
LM-MLEM: List-Mode Maximum Likelihood Expectation Maximization
GNB: Gaussian Naive Bayes
ROC: Receiver Operating Characteristic [curve]
AUROC: Area Under Receiver Operating Characteristic [curve]
GTV: Gross tumor volume

## Code availability

All of the simulation, analysis and reconstruction scripts used in this work are available from the corresponding authors upon reasonable request.
