# Supplementary material for: A hybrid multi-particle approach to range assessment-based treatment verification in particle therapy
Source: Sci Rep. 2023 Apr 25;13:6709. doi: 10.1038/s41598-023-33777-w (PMC10130067; doi:10.1038/s41598-023-33777-w)
Supplement: Supplementary file 1 — Supplementary Figures. [file 41598_2023_33777_MOESM1_ESM.pdf]

A hybrid multi-particle approach to range assessment-based treatment verification in particle therapy - Supplementary information

Meric, I., et. al.

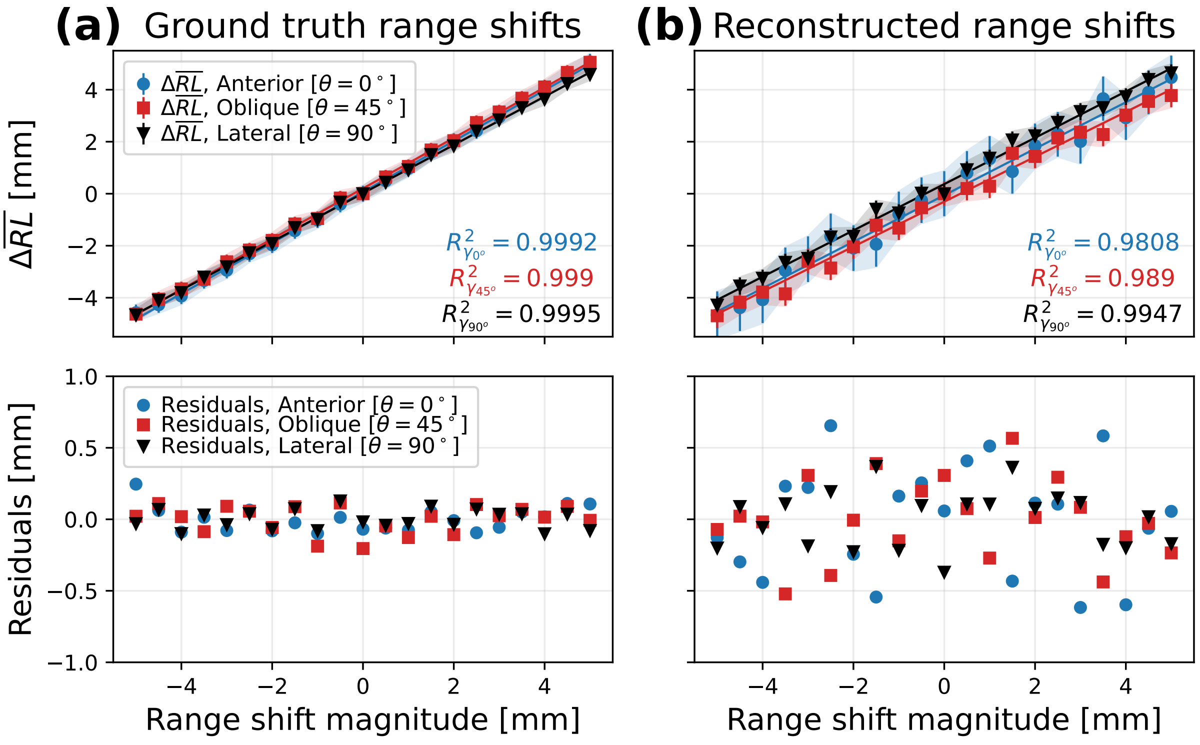

FIG. 1. The correlation between the true range shifts in mm and the mean of the calculated range shifts, $\Delta\overline{RL}$, using **(a)** the true production distributions, and **(b)** the LM-MLEM reconstructed production distributions of PGs in the patient model. Both sub-figures show the results obtained for a proton intensity of 10^8. Also shown (in the bottom panels) are the residuals from a linear best fit to the data points. A strong correlation is observed for all three orientations of the NOVCoDA.

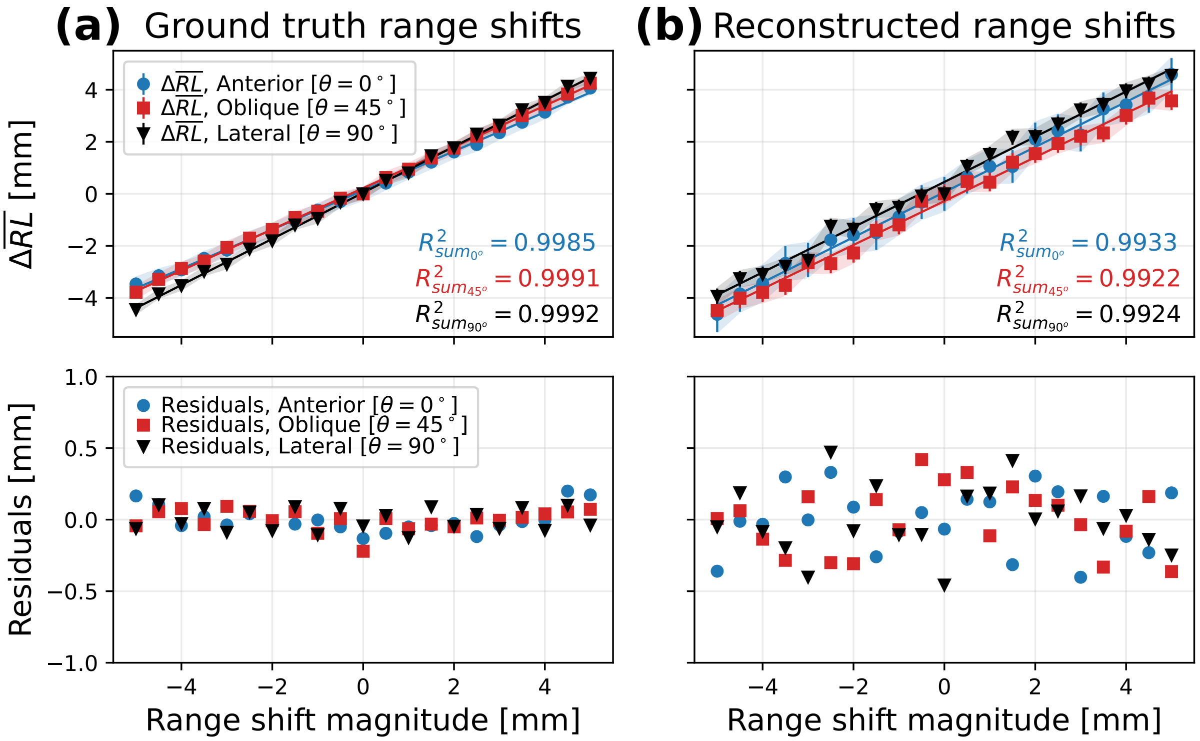

FIG. 2. The correlation between the true range shifts in mm and the mean of the calculated range shifts, $\Delta\overline{RL}$, using the sum of **(a)** the true production distributions, and **(b)** the LM-MLEM reconstructed production distributions of PGs and FNs in the patient model. Both sub-figures show the results obtained for a proton intensity of 10^8. Also shown (in the bottom panels) are the residuals from a linear best fit to the data points. A strong correlation is observed for all three orientations of the NOVCoDA.

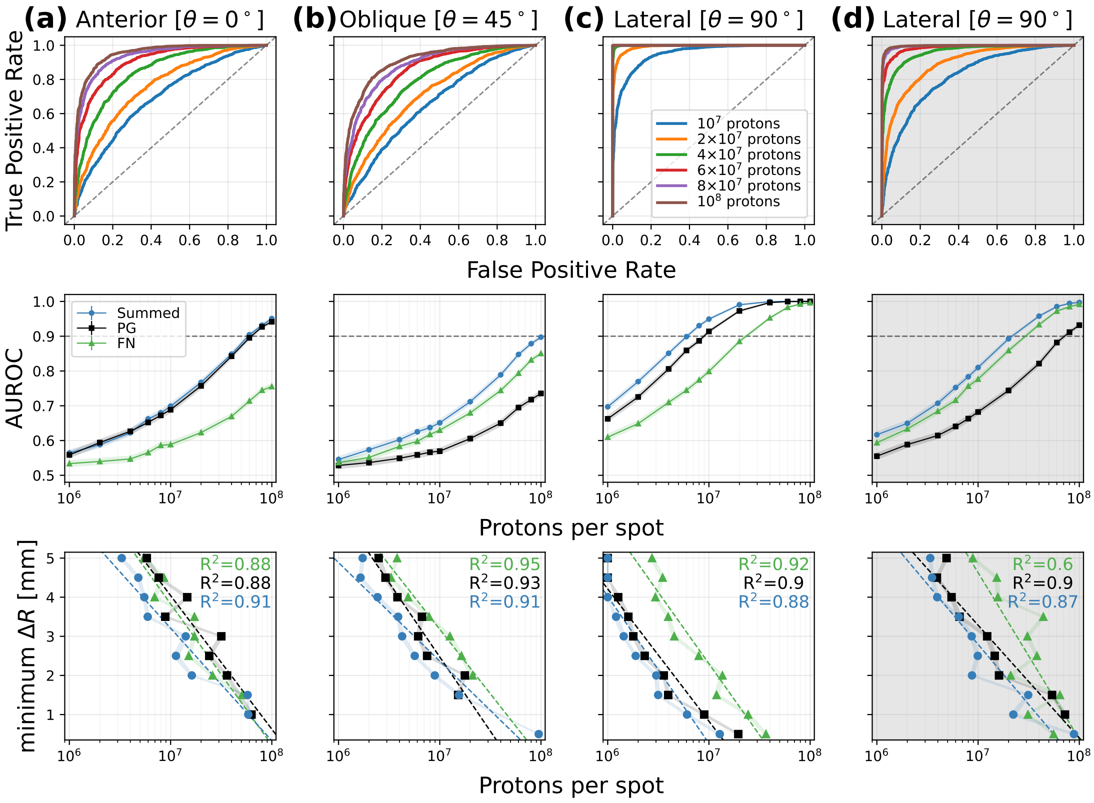

FIG. 3. Receiver Operating Characteristic (ROC) curves and average Area Under ROC ($\overline{\textbf{AUROC}}$) curves for a range shift of 1.0 mm and minimum detectable range shifts. The upper panel shows the ROC curves evaluated at a range shift magnitude of 1.0 mm for the reconstructed FN and PG sum data-set. The middle panel shows the calculated $\overline{AUROC}$ values. The lower panel shows the minimum detectable range shifts as a function of proton intensity. Each of these are shown for a NOVCoDA orientation of $\theta = 0°$ **(a)**, $\theta = 45°$ **(b)** and $\theta = 90°$ **(c)**, as well as the $\theta = 90°$ case with the estimated effects of time, energy, and position resolutions (and segmentation of a realistic detector) included in **(d)**. The uncertainties in terms of one standard deviation are shown as bands around the $\overline{AUROC}$ values in the middle panel.

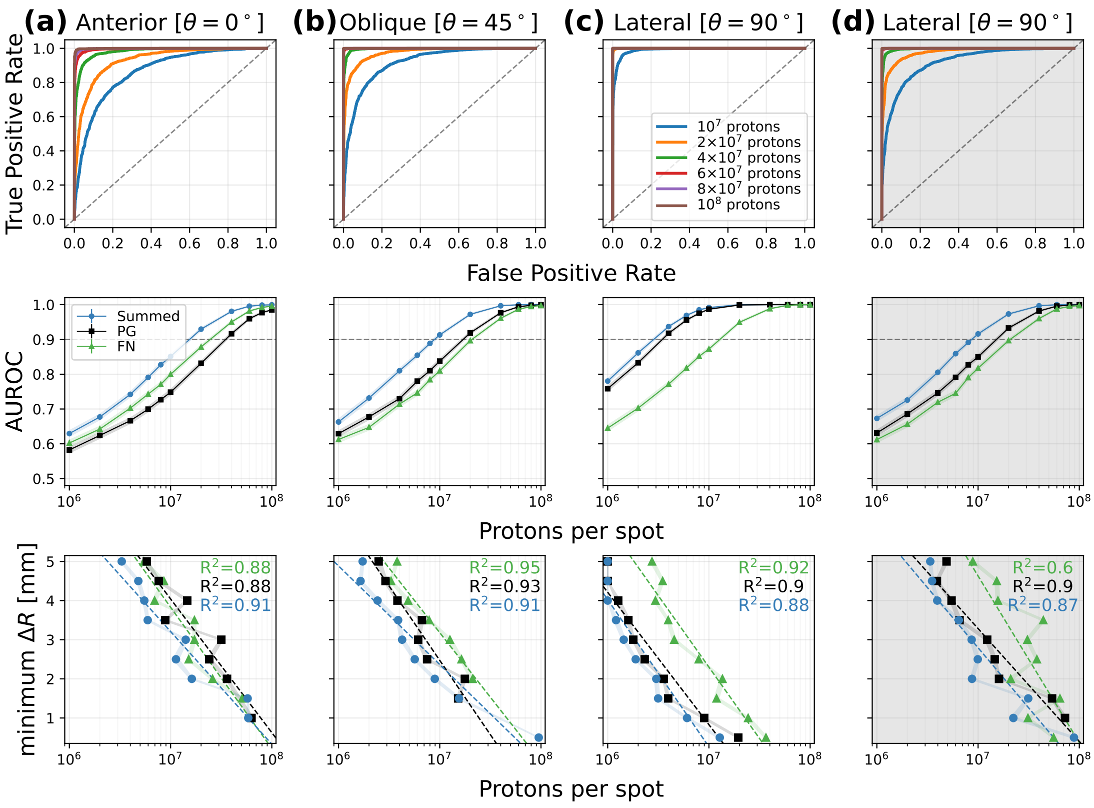

FIG. 4. Receiver Operating Characteristic (ROC) curves and average Area Under ROC ($\overline{\textbf{AUROC}}$) curves for a range shift of 2.0 mm and minimum detectable range shifts. The upper panel shows the ROC curves evaluated at a range shift magnitude of 2.0 mm for the reconstructed FN and PG sum data-set. The middle panel shows the calculated $\overline{AUROC}$ values. The lower panel shows the minimum detectable range shifts as a function of proton intensity. Each of these are shown for a NOVCoDA orientation of $\theta = 0°$ **(a)**, $\theta = 45°$ **(b)** and $\theta = 90°$ **(c)**, as well as the $\theta = 90°$ case with the estimated effects of time, energy, and position resolutions (and segmentation of a realistic detector) included in **(d)**. The uncertainties in terms of one standard deviation are shown as bands around the $\overline{AUROC}$ values in the middle panel.

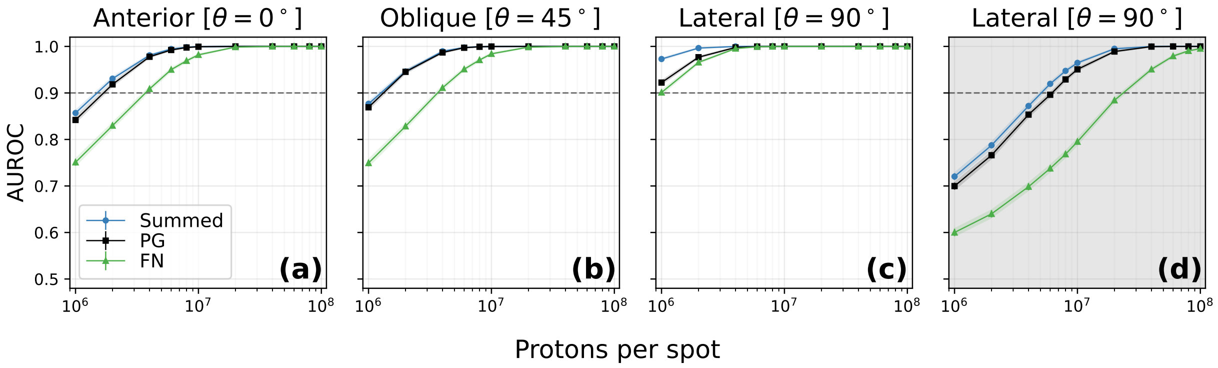

FIG. 5. $\overline{\textbf{AUROC}}$ curves for the clinical case at three different orientations of the NOVCoDA with respect to direction of the incident proton beam prepared using the ground truth production distributions of FNs, PGs and their sum. The figure summarizes the calculated $\overline{AUROC}$ values using only FN and only PG distributions as well as for the sum of FN and PG distributions for an orientation of $\theta = 0°$ **(a)**, $\theta = 45°$ **(b)** and $\theta = 90°$ **(c)**, as well as the $\theta = 90°$ case with the estimated effects of time, energy, and position resolutions (and segmentation of a realistic detector) included in **(d)**. Statistical uncertainties in terms of one standard deviation of the $\overline{AUROC}$ are also shown explicitly as a narrow band around each curve.

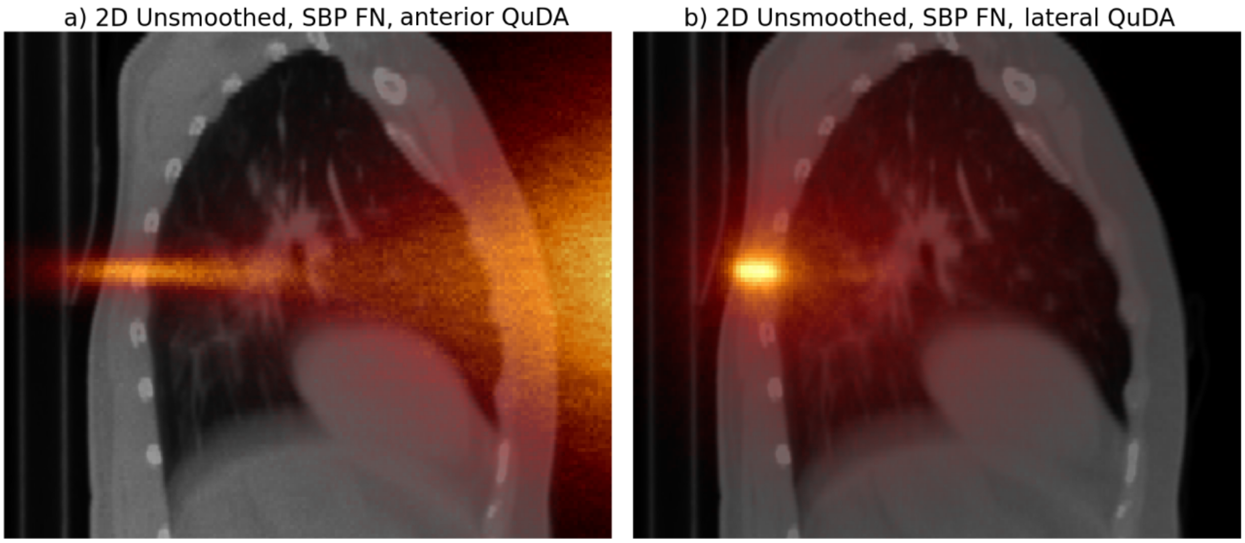

FIG. 6. Reconstructed FN distributions from unsmoothed simple back projection (SBP) of event cones for the clinical example for **(a)** the anterior ($\theta = 0°$) and **(b)** the lateral ($\theta = 90°$) NOVCoDA orientations. The blurring artifacts, that are also common to Compton imagers, are clearly visible for the anterior ($\theta = 0°$) NOVCoDA orientation.

FIG. 7. A gif animation of the erosion/dilation processing of the CT-based patient model. Please see the corresponding supplementary file.
