# Supplementary figures and images for: A hybrid multi-particle approach to range assessment-based treatment verification in particle therapy
Source: Sci Rep. 2023 Apr 25;13:6709. doi: 10.1038/s41598-023-33777-w (PMC10130067; doi:10.1038/s41598-023-33777-w)

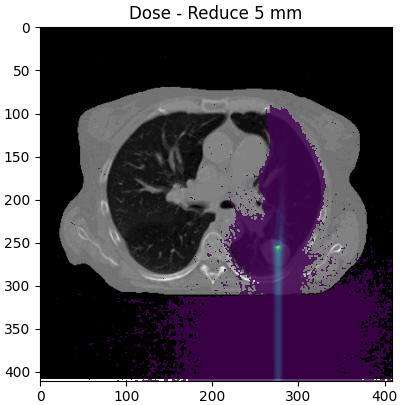

Supplement: Supplementary file 2 — Supplementary Figure 7. [file 41598_2023_33777_MOESM2_ESM.gif]
